# Therapeutic Use of Antitoxin

**Published:** 1895-04

**Authors:** J. H. Huddleston


### III. Therapeutic Use of Antitoxin.

BY J. H. HUDDLESTON, M.D.

When the blood is drawn it passes into sterilized flasks, and is then kept in the refrigerator in the laboratory three or four days, to give time for the clot to squeeze out the serum.

*Testing the serum.* The serum obtained, the next thing is to test its strength, *i. e.*, to find out how much antitoxin it contains. In doing this the method originated by Behring is followed. The toxin bouillon is again taken, and the amount of it necessary to kill a guinea-pig weighing two hundred and fifty grammes is carefully ascertained by experiment. Ten times this amount of the toxin bouillon is then mixed with a certain amount of the serum to be tested, and the mixture is injected into a guinea-pig. If the pig dies there is evidently not enough of the serum in the mixture; more is added and the injection repeated. Thus, by a series of experiments it is possible to find out just how much serum must be mixed with ten times the fatal dose of the toxin to keep the pig from dying. Serum of which one-tenth of a cubic centimetre is sufficient to save the guinea-pig is called by Behring the normal antitoxin serum, and one cubic centimetre of this serum is called one antitoxin unit. The samples now taken from the different horses under treatment vary from 300 to 2000 units, *i. e.*, $\frac{1}{2000}$ of a cubic centimetre of the strongest serum would save a guinea-pig weighing 250 grammes if injected with ten times the fatal dose.

*Bottling.* The tested serum is put up in sterilized bottles provided with rubber sterilized stoppers. Special bottles are now being designed, which are to be of two convenient sizes (10 c.c. and 20 c.c.) and to serve as measures of the serum. Various antiseptics have been tried—carbolic acid, chloroform, trikresol, thymol, and camphor, and at present the custom is to put a small piece of sterilized camphor in every bottle Thus prepared, a bottle will (if kept in a dark cool place) maintain its strength probably for six months. If not kept with sufficient care it may become turbid, and in that case its use is not to be recommended. It has been found, however, that thymol used as an antiseptic causes in the serum a chemical change which is evident as turbidity.

*Syringe.* To make the injection there have been proposed various syringes, none of which can claim absolute superiority over all the rest. One essential in a syringe is that it be so constructed that it can be readily disinfected. A glass barrel and a piston with asbestos packing best serve this end. The barrel should be large enough to hold the entire dose, and the needle should be long enough to deposit the fluid well under the skin. The serum is viscid, and the calibre of the needle must, therefore, be rather large.

*Dose.* The dose varies, of course, with the strength of the antitoxin. A month ago, with the comparatively weak antitoxin then produced, 25 to 30 cubic centimetres were injected. At present 10 to 12 c.c. suffice for a child four or five years old, and proportionately less for a baby. For immunization 3 or 4 c.c. are usually thought sufficient.

*Number of injections.* In the majority of cases one injection is all that is given. Not very frequently, however, a second injection is given twelve to eighteen hours after the first, and three, four, and even seven injections have been given at similar intervals.

*Precautions at injection.* The skin is carefully cleansed with water, and soap if necessary, 5 per cent. carbolic acid solution, and alcohol. A fold of the skin is then pinched up with clean fingers, and the needle pushed in parallel with the subjacent muscle an inch or more, and then the serum is slowly injected. The thighs, buttocks, and lumbar muscles are the favorite sites for injection. Some pain, of course, attends the prick, and some, too, though surprisingly little, the distention of the parts by the serum.

*Results of injection.* These vary very markedly with the duration of the disease before injection. If the injection is given on the first day there is usually a prompt fall of temperature and pulse and an improved general condition. The same is true, to a less marked degree, on the second, third, and, perhaps, the fourth day; but after the fourth the antitoxin seems very frequently to have but little effect on the constitutional symptoms. On the local condition, on the contrary, the antitoxin seems to have very constantly the effect of diminishing the engorgement and the discharge and of loosening the membrane, provided only that the child lives more than a few hours after the injection. In "croup" cases, *i. e.*, in all cases in which the larynx is involved, there is just as much need of intubation as ever, but with the tube inserted there is a marked decrease in the mortality, and even in the fatal cases there is a very notable prolongation of life over what is customary.

The mortality in "croup" cases at the Willard Parker Hospital was never, in pre-antitoxin times, much below 50 per cent., but in the last eighteen croup cases treated by antitoxin there were only four deaths. The mortality in severe non-laryngeal cases here in New York varies, but is probably not much less than 30 to 40 per cent. in children under five years. With the antitoxin treatment the mortality, as far as we now know, is only about 15 per cent. The results of the immunization treatment are not yet known, but there are two cases on record which received, one a large immunization injection (18 c.c.) and the other a small one (4 c.c.), and both became ill with diphtheria a month after injection. It may be inferred, therefore, that the immunization conferred does not last more than a month. Among the minor effects of antitoxin it may be mentioned that the albuminuria present before injection has been found in some cases to diminish very markedly after injection. In a certain proportion of cases a general erythema, often accompanied by urticaria, appears in from ten to fourteen days after the injection, and lasts from a few days to two weeks, finally disappearing without scaling. Rarely a well-marked purpura has been seen; neuralgia in the limbs, hyperæsthesia of the skin, and great weakness have all been mentioned as occasionally coming on after the injection, and lasting usually only a very short time. It is proper to remark that well-marked nephritis has appeared, and that in a number of cases late death has occurred from heart failure, just as happens in diphtheria not treated by antitoxin.

Finally, a rough summary of the results of the antitoxin treatment here in New York would be: Out of every hundred severe cases of diphtheria in children under five years twenty more are saved by the antitoxin treatment than are saved by any other. Old complications are not made worse or more frequent, and no new complications, except eruptions on the skin, are introduced.

---

The prospect of lifting the embargo against American cattle and live stock products by Germany grows brighter, as the opinions of the foreign veterinarians are being more thoroughly diffused.

